# Radium

**Published:** 1906-07

**Authors:** F. G. Hodgson

**Affiliations:** Atlanta, Ga.


### RADIUM.*

BY F. G. HODGSON, M.D.,

ATLANTA, GA.

The action of X-rays in rendering bodies transparent was first described by Roentgen in 1895. This was followed by the discovery of certain rays given off from the metal uranium by Becquerel in 1896. These discoveries were much talked of by the scientists, and Madam Curie in Paris became especially interested and began her investigations. She found that pitchblende, composed chiefly of uranium and thorium, gave off Becquerel rays. She separated the uranium from the pitchblende and found that the remaining mass was more radio-active than the uranium. Then, with the aid of her husband, Prof. Curie, she continued her experiments, reducing the mass to simpler com-

* Read before the Fulton County Medical Society, March 15, 1906.

pounds, each time selecting the most radio-active portion until they finally separated a very active substance in the form of a chloride, to which they gave the name "radium." The element itself has not been isolated, but Mme. Curie calculated its atomic weight to be 225, thus showing it to be one of the heaviest metals known, and placing it in the group with magnesium, calcium, strontium and barium, according to the periodic law. Mme. Curie took two tons of pitchblende and after several months of repeated precipitation, filtration, crystallization and decantation, obtained about 1-10 grain of radium chloride—just 1-10 grain from over 4,000 pounds; this is rarer than the gold in sea water.

*Occurrence:* Radium is found chiefly in association with uranium in the pitchblende found in Bohemia and a few places in Germany. The supply is very limited, the immense difficulty of isolation and the skill required make it one of the most expensive substances known. The increased demand has also raised the price considerably; 1 gram would be worth about $80,000. Some medicinal springs and baths are reported to contain radium or radio-active substances to account for their wonderful healing properties, but they contain only very minute quantities. Careful search is being made in this and other countries for radium bearing ores, but so far with no result.

*Physical and Chemical Properties:* The bromide of radium is the salt generally used. It occurs as small white crystals; is soluble in water. It gives a carmine color to a Bunsen flame. It has a characteristic spectrum. Its properties are almost identical with the barium salt. It gives off *heat* continuously without any appreciable change or diminution. One gram maintains itself 5° C. above the surrounding air; 1 gram emits energy equal to about 100 gram-calories per hour. Radium salt in solution will after a short time give off the same quantity of heat as the dry salt. The most interesting property of radium is the spontaneous giving off of rays known respectively as the X, B and Y-rays.

*X-rays* are very minute particles of matter, about twice the mass of an hydrogen atom and travelling at the enormous rate of 25,000,000 meters, or 15,500 miles, per second. You can imagine that the momentum of these particles, though minute, must be considerable. They are not very penetrating, being stopped by a thin mica plate or glass container. The particles are positively charged electrically, and will discharge an electroscope. They may be deflected by a magnet toward the negative pole.

*B-rays* are still more minute particles of matter, being only 1-1000 the mass of an hydrogen atom. They travel even more swiftly, traversing about 150,000,000 meters or about 100,000 miles per second—this approaches the swiftness of light. If unimpeded, a particle would travel about four times around the earth in a second. These particles are negatively charged with electricity, and are deflected by a magnet in the opposite direction and more strongly than the X-rays. They are more penetrating, easily passing through a glass container, and penetrating the skin for about half an inch. They are considered to be identical with the cathode rays of a Crooke's tube. They are sometimes called corpuscles or electrons. They are probably the most valuable therapeutically, and the burning of the skin is due to them.

Y-rays are probably not particles of matter at all, but are undulations of ether. They resemble very closely the X-rays given off from a hard or high vacuum tube. They travel with enormous velocity and are very penetrating, they will pass through a foot of solid iron. Bones offer practically no resistance to them. They do not discharge an electroscope, and are not deflected by a magnet.

Ninety-five to ninety-nine per cent. of the radiant energy is due to the X-rays. A very minute effect is due to the Y-rays, leaving about 1 to 4 per cent. due to the B-rays. The bulk of the therapeutic effect, however, is due to the B-rays, for the X-rays are absorbed by the glass container.

When we consider the minute quantities of radium which are used experimentally (usually a fraction of a grain, and that impure) it is easy to let the imagination play as to what could be done with large quantities. It has been suggested that with a pound the house could be heated and the cooking done for a large family for generations; or with a sufficient quantity a transatlantic steamer could be driven back and forth across the ocean indefinitely.

The combined effects of these radiations are:

1. *Fluorescence and Luminosity.* This is beautifully shown in a little instrument invented by Crookes and known as the spinthariscope. This consists of a cylindrical brass tube, about an inch in diameter by three inches long. At one end is a circular fluorescent screen of calcium fluoride or zinc sulphide, and in front of this a very minute particle of radium on a moveable arrow. At the other end of the tube is an adjustable lens. Upon going into a dark room and focussing the lens upon the screen one can see minute sparks flying off in every direction resembling a miniature shower of shooting stars. This is due to the X-rays impinging upon the screen. Phosphorescence can also be observed with any fluoroscope, but you only get a light and not the sparks observed in the spinthariscope. Diamonds, barium-platino-cyanide, willemite, kunzite and zinc sulphide are all made phosphorescent.

2. *Coloration of Bodies.* Diamonds change color and glass is turned blue in the prolonged presence of radium.

3. *Chemical and Photographic Effects.* Paper becomes brittle and turns dark after prolonged exposure. Ozone is formed in the air around radium. The rays are in no way affected by extreme heat or cold. A photographic plate is darkened by the rays even through considerable thickness of dark paper; in this manner the image of various articles can be reproduced on the plate similar to those made by X-rays, but there is not the same sharpness of outline, and bones can not be outlined.

4. *Physiological and Pathological Effects.* Seeds exposed to radium will be stunted in growth or killed, depending upon the length of exposure. It takes about a week to kill the mustard seed. Small mice and young guinea pigs after several hours exposure show nervous weakness, then paralysis and finally death—their hair will fall out and may return gray, as was shown by Roux in Paris. Human skin is burned, many pathological growths are effected, and skin lesions cured—but this will be discussed later.

5. *Electrical Effects.* The rays will discharge an electroscope, due to the ionization of the surrounding air. If the spark gap of an induction coil is so arranged that the spark will not quite jump across, then some radium be brought near the coil, the spark will jump across easily.

Radium, besides giving off continually heat and three different kinds of rays, has also another remarkable property; it gives off a *gaseous emanation.* This can be collected by putting some radium in a glass bulb and connecting it with another bulb by means of a glass tube. The emanation is given off more rapidly by heating or dissolving the radium in water and passing air through it. After the emanation is driven off into the other bulb it can be examined. It is found to be temporarily radio-active and has the power of inducing temporary activity in other bodies. The radium loses some of its radio-activity when so treated, but after a short time it regains its normal strength, and, strange to say, it regains its activity at exactly the same rate that the emanation loses its activity.

This emanation is temporarily radio-active, and may cause phosphorescence in bodies and discharges an electroscope. It acts like a gas, in that it may be condensed by exposure to the extreme cold or liquid air. After the lapse of several weeks a most wonderful and unheard of transformation occurs—it has been shown by the noted chemist Ramsay that this emanation of radium has been transmuted into another element, helium, and gives the characteristic spectrum of that element. If this be true, it is the only instance known to man in which one so-called element has been converted into another; this makes the hope of the old alchemists seem a real possibility.

The amount of radio-activity of a specimen of radium is based upon the radio-activity of an equal mass of uranium used as an unit. Specimens vary in activity from 240 to 1,500,000 units. The activity of a specimen immediately after preparation is only about one-fourth its final value; it increases in strength for a month when kept dry in a closed container. The activity is determined in several ways, e. g.:

1. The rapidity with which it discharges an electroscope, due to the ionization of the surrounding air.

2. The effect upon a photographic plate enclosed in dark paper.

3. The way it illuminates a phosphorescent screen.

Luminosity in the dark is not a test of activity—very impure specimens may give off considerable light.

*Induced or Excited Radio-activity.* A large number of bodies when left directly exposed to the radium salt become temporarily radio-active. This does not occur when the radium is enclosed in a sealed tube, and is due to the effect of the emanation. So a tube of radium placed in a solution does not render that solution active. Many fake radium cures are manufactured in this way, and many of the so-called radium cancer cures sold at enormous prices have never been near radium. The amount of radio-activity induced upon a given area is independent of the substance acted upon. All induced radio-activity decays very rapidly, falling to half its value in thirty minutes, so it can not be of very much practical value to physicians.

The source of the continued and apparently undiminishing energy has been subject to much discussion. The most generally accepted view is that it is derived from a slight disintegration of the radium atom, which must then be very complex. Others say it is due to electric currents around the atom. A few hold that it must get its energy from some external source. Recent observations tend to show that the sun must contain radium to account for the amount of heat given out and the slow rate of cooling of that body; also that the quantity of radium in the earth accounts for its present heat, in spite of the ages it has been in cooling.

### ACTION OF RADIUM UPON LIVING TISSUES.

*Gross Effects.* Take, for example, a specimen of pure radium bromide, rated about 1,500,000 activity, and place it upon the skin in one place for five minutes, another place half an hour, another two hours; there will be no immediate effect upon the skin. After about two weeks the five-minute spot will turn red and remain in that condition about two weeks and gradually get well, any hair present will fall out. The half-hour spot will begin to be red sooner, may go on to vesication, but will heal in about a month. The two-hour spot will take effect still sooner, will then vesicate and may go on to ulceration, and may take two months or more to heal and leave an atrophic looking skin. Prof. Curie carried a specimen in his vest pocket for several hours, and as a result had a bad ulcer which took several months to heal. So according to the length of exposure we get erythema, dermatitis, vesiculation, ulceration and necrosis. Any hair present will fall out, but will return if the burn is not too deep. After healing there may be no scar, no atrophy, or pigmentation, or even agiectiasis has been reported. So we see the results on the normal skin closely resemble those of the X-rays.

Radium produces a sensation of light in eyes that are partially blind, but no effect when the retina is destroyed. This gave rise to the report that radium would cause the blind to see.

Ova and embryos of young frogs and other animals were exposed to radium for varying lengths of time; after a latent period cell division was inhibited and differentiation and growth was retarded, just as was the case with the seeds—the rays seem to have a selective action on young and rapidly growing cells.

*Microscopical Effects.* In a patient to be operated upon for carcinoma of the breast Dr. Robert Abbe applied radium to the sound skin, to the cancerous area, and in one place buried the radium under the skin within the tumor for several hours. After amputation the breast was given to Dr. F. C. Wood, and he reported as follows:

1. On the skin a superficial necrosis always takes place, the longer the application the deeper the devitalization of the cells.

2. Leucocyte infiltration shows around all vessels and nerves.

3. Where an area of cancer underlies the skin a softening and devitalization of the central cells of nests nearest the radium takes place.

4. Where the radium was thrust into the cancer an area of complete destruction of cancer cells for not more than one-fourth inch takes place. A recurrent nodule of cancer was exposed to strong radium twenty times during three months and diminished to one-third its former size. It was then excised, and upon examination showed marked fibrous hyperplasia among the cancer cells. Abbe says that those malignant cells which escape destruction or retrograde change show a striking quiescence which may mean the death of the vital force which makes them malignant. In this connection we can not fail to be reminded of the seeds exposed to radium which fail to germinate.

*Bactericidal Effect.* Radium has been found to kill various germs. B. typhosus; B. of cholera; B. prodigeosus and others were killed. The radium must, however, be applied very close (½mm.) and for at least twenty-four hours. It is of no practical value in cleansing an infected wound. Radium burns are, however, very resistant to infection, so radium seems to increase the resisting power of the cells.

Various physiological effects are reported, e. g., red blood cells give up their hemoglobin more readily to solution, oxyhemoglobin is converted into methemoglobin. The digestive ferments, emulsin and trypsin, are rendered inert. Vaccine is rendered inefficient. Various toxins become innocuous.

*Therapeutic Value.* Radium has been found to be of value mainly along the same line of cases as the X-rays. This was to be expected from the similarity of effect upon the healthy skin. It has been used with best results in cases of lupus vulgaris, superficial cancerous growths, and various skin lesions. I have collected from various sources the following cases and results of radium treatment:

Williams, in the *Medical News,* reports:

Mammary Cancers: None cured, 3 improved, 1 unimproved.

Epidermoid cancers: 11 cured, 12 improved.

Rodent ulcers: 2 cured, 3 improved.

Lupus vulgaris: 4 cured.

One keloid, 1 psoriasis, 2 chronic eczemas, 9 acne vulgaris improved or cured.

McLeod, in the *British Medical Journal,* reports:

Two lupus vulgaris cured, 1 lupus verrucosa improved, 1 rodent ulcer cured.

Exner, of Vienna, reports marked improvement in a melano-sarcoma, temporary improvement in symptoms and increase in calibre of three cases of cancer of the esophagus.

Max Einhorn, of New York, reports nine cases of cancer of the esophagus treated by radium. The radium was introduced in a capsule on the end of a flexible bougie and held in contact with the stricture. He calls this instrument a radiodiaphane.

Of the nine cases so treated, six show increase in calibre of the esophagus and greater ease in taking food. One patient improved markedly in weight. In three cases no improvement was noted, due to irregularity in treatment or to insufficient time having elapsed. Einhorn does not claim to cure these cases, but any relief to such hopeless cases is to be welcomed as a step in advance.

Lassar, of Berlin, is very enthusiastic in his praise of radium in the cure of certain skin lesions, but Bulckley, of New York, thinks X-rays are just as efficient.

Ordinary warts are easily removed by one to three exposures of one-half hour each. Port wine marks will become whiter after exposure and angiomata have been cured.

Abbe, of New York, reports some excellent results from radium treatments. Cases of lupus, rodent ulcer, epithelioma, verruca and sarcoma have been cured by him. Also some improvement in recurrent cancerous nodules and inoperable cancer of the cervix uteri. A remarkable case of his was a giant cell sarcoma of the lower jaw about the size of a walnut. He thrust the radium into the mass on several occasions and it began to diminish in size and finally disappeared and has remained cured for about two years. He also had marked improvement in a large exophthalmic goitre by plunging the radium tube into the goitre. The tumor at the end of a month was reduced to half its size, and other symptoms improved.

Pusey, of Chicago, sums up the indications for radium as:

1. Inflammatory diseases of the skin as eczema, psoriasis, lichen planus.

2. Bacterial diseases of the skin: Acne, sycosis, lupus, blastomyces.

3. To cause destruction of tissues of low resistance: Carcinoma, sarcoma, lupus and warts.

The *London Lancet,* March 26, 1904, reports two cases of tuberculous ulcers of the throat cured. It also recommends radium for tuberculosis of the superficial bones of the hand and foot, sternum and ribs.

Beck, in the *Chicago Medical Record,* December 15,, 1905, reports cures in the case of tuberculous ulcer of the septum nasi, and a case of sycosis. Relief to pain in a case of sarcoma pharyngis. Pain was also relieved in neuralgic cases, and marked relief to the pains of tabes dorsalis. A secondary tuberculosis of the larynx and cancer of the larynx and cervical glands were treated without result.

Czerney's clinic in Berlin reports in the *Deutsch Med. Wochens,* Vol. XXX, No. 42, 2 naevi, 5 angioma, 7 lupus, cured; 5 carcinoma and 1 melano-sarcoma not cured.

*Munchner Med. Wochens,* Vol. LII. No. 34: Bacteria are killed by exposure to radium, and their toxins are rendered inert. This was proved by injections into rabbits.

*Mitteilung aus den Grenzegebieten,* Vol. XIV, No. 5: Two large angiomata were cured by one-hour exposure. In two weeks a scab formed, after three weeks there was a pink scar, and after six weeks the skin appeared normal.

No spermatozoa were found in the testicle or epididymus of a guinea pig after radium had been attached to that organ for twenty-four hours.

*Riforma Medica,* Naples, Vol. XXI, No. 15: Tizzoni reports that animals inoculated with rabies would recover after exposure to radium, while controle animals would die. He also says that the rabies virus exposed to radium was rendered innocuous and, more than that, it could be converted into a vaccine.

Robarts, in the *Journal of Physical Therapy,* February, 1906, reports two cases of bleeding piles and three cases of fissure in ano cured by radium and dilatation. My own experience has been very limited; I have removed several warts by half-hour exposure, causing no pain and leaving no scar. I have under treatment a beginning swelling of the thyroid in a young girl, which has diminished slightly under three applications, but it is too early to make a report on this case. I have noted the retardation of growth in mustard seeds exposed to the rays, and have confirmed a number of the photographic, phosphorescent and electrical phenomena.

*Method of Use.*—On the external parts the radium is applied directly to the affected part in a small glass tube, the surrounding healthy parts being protected by a thin layer of lead. The tube may be sterilized and inserted into tumors. A large variety of applicators are in use for treating the various mucous cavities. The length of exposure varies from twenty minutes to twenty-four hours inside tumors, the average exposure is one-half to one hour. These are repeated two or three times a week, if necessary.

*Conclusions.*—In radium we have a substance of immense interest to both scientists and the medical profession. Its action resembles closely the X-rays, but it has the advantage of the ease with which it can be carried around and used. It is of especial use in the mucous cavities, such as the nose, mouth, throat, esophagus, vagina, uterus and rectum. It often succeeds where the X-rays have failed. It is of undoubted value in lupus, rodent ulcer, epithliomata, warts, and some cases of sarcoma, and many forms of skin disease. It is a powerful little agent, and has performed some wonderful cures. It should be given a thorough trial. If larger quantities can be procured and its cost diminished it will surely be of great service to mankind.

REFERENCES.

Mme. Curie. Researches sur les substances radio-actives; Paris, 1905.

Soddy. Lectures on Radioactivity, *Electrician*, October, 1903; February, 1904.

Sir O. Lodge. *Archives of the Roentgen Rays*.

Halkin. *Archiv. fur Dermatol. und Syphillog.* 1903.

Rollins. *Boston Medical and Surgical Journal*, December, 12, 1903.

Williams. *Medical News*, February 6, 1903. *Boston Medical and Surgical Journal*, February 25, 1905.

McK. Davidson. *British Medical Journal*, January 23, 1904.

Plummer-Darier. *London Lancet*, April 16, 1904.
Goodspead. *Archiv. Electroland Radiol*, March and April, 1904.
Rutherford. Radioactivity. Cambridge, Eng., 1904.
Danne. Le Radium: sa preparation et ses proprieties. Paris, 1904.
Piffard. *Medical Record*, June 18, 1904, and April 2, 1905. *Journal of Cutaneous Diseases*, June, 1904.
Pusey. Radium and Its Therapeutic Possibilities. *Journal American Medical Association*. July 16, 1904.
Morton. Treatment by Radium Rays. *British Medical Journal*, April 23, 1904.
McIntyre. Recent Electrotherapy. *British Medical Journal*, April 23, 1904. and December 12, 1903.
Turner. *British Medical Journal*, December 12, 1903.
Abbe. Radium and Radioactivity. *Yale Medical Journal*, June 1904, and *Medical Record*, June 4, 1904. The Subtle Power of Radium, *Medical Record*, August 27, 1904. Exophthalmic Goitre Reduced by Radium, *Archives of Roentgen Rays*, etc., March, 1905.
Heinecke. *Deutsch Medical Wochenschrift*, August 4, 1904.
Schaper. *Deutsch Medical Wochenschrift*, September 29, 1904.
Wohlgemuth. *Berlin Klin. Wochenschrift*, June 27, 1905.
Duncan. The New Knowledge. Barnes & Co., 1905.

### DISCUSSION ON DR. HODGSON'S PAPER.

*Dr. Wolff* complimented the speaker upon the thoroughly prepared paper. He said that two years ago he was very much interested in the subject, and procured a specimen of Cúrie radium of 7000 activity. With this weak specimen his results were negative.

*Dr. Hutchins* said he did not think the results obtained would justify the great expense of procuring a specimen of sufficient activity to get good results. He said that there were other therapeutic measures in use that were just as good.

*Dr. Hodgson* replied that he thought that the number of remarkable cures by radium were sufficient to give it a much more extensive trial, and that further use would show it to be of still greater value.

---

Do not be too sure that a mass in the region of the pylorus is a carcinoma. In some cases the infiltration around a chronic ulcer is very extensive and may simulate the feel of a new growth.—*American Journal of Surgery*.

